# Bis­(chloro­acetato-κ*O*)bis(trimethyl­silylmethyl)tin(IV)

**DOI:** 10.1107/S1600536811028649

**Published:** 2011-08-02

**Authors:** Rui-Fang Ding, Qi-Bao Wang

**Affiliations:** a Department of Pharmacy, Jining Medical College, Xueyuan Road 669, Rizhao, People’s Republic of China

## Abstract

In the title complex, [Sn(C_2_H_2_ClO_2_)_2_(C_4_H_11_Si)_2_], the Sn^IV^ ion is coordinated in a distorted tetra­hedral environment formed by two O atoms from two monodenate chloro­acetato ligands and two C atoms from two trimethyl silyl ligands. Two further weak intra­molecular Sn⋯O contacts [2.744 (2) and 2.655 (2) Å] are formed by the chloro­acetato ligands.

## Related literature

For a related structure, see: Parvez *et al.* (1997 ▶).
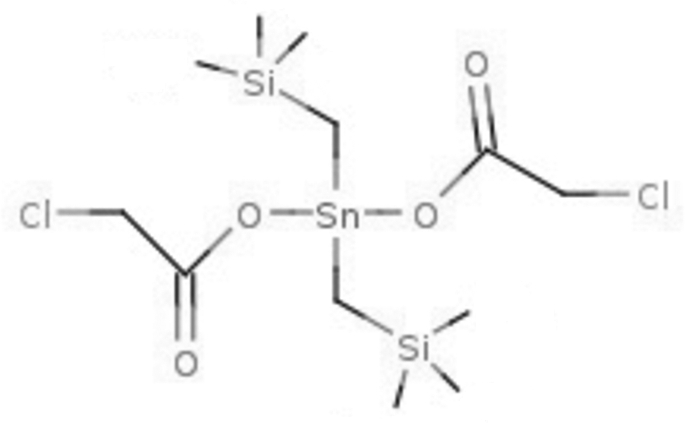


## Experimental

### 

#### Crystal data

[Sn(C_2_H_2_ClO_2_)_2_(C_4_H_11_Si)_2_]
*M* *_r_* = 480.10
Triclinic, 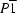

*a* = 10.258 (3) Å
*b* = 10.767 (3) Å
*c* = 10.808 (3) Å
α = 71.529 (2)°
β = 88.733 (3)°
γ = 74.457 (3)°
*V* = 1088.2 (5) Å^3^
*Z* = 2
Mo *K*α radiation
μ = 1.54 mm^−1^
*T* = 293 K
0.28 × 0.22 × 0.17 mm

#### Data collection

Bruker SMART CCD diffractometer
Absorption correction: multi-scan (*SADABS*; Bruker, 1997) ▶ *T*_min_ = 0.673, *T*_max_ = 0.780
6849 measured reflections
3792 independent reflections
3311 reflections with *I* > 2σ(*I*)
*R*_int_ = 0.029

#### Refinement

*R*[*F* ^2^ > 2σ(*F* ^2^)] = 0.032
*wR*(*F* ^2^) = 0.089
*S* = 1.04
3792 reflections
197 parameters
H-atom parameters constrained
Δρ_max_ = 0.66 e Å^−3^
Δρ_min_ = −0.47 e Å^−3^

### 

Data collection: *SMART* (Bruker, 1997) ▶; cell refinement: *SAINT* (Bruker, 1997) ▶; data reduction: *SAINT* ▶; program(s) used to solve structure: *SHELXS97* (Sheldrick, 2008 ▶); program(s) used to refine structure: *SHELXL97* (Sheldrick, 2008 ▶); molecular graphics: *SHELXTL* (Sheldrick, 2008 ▶) and *PLATON* (Spek, 2009 ▶); software used to prepare material for publication: *SHELXTL*.

## Supplementary Material

Crystal structure: contains datablock(s) I, global. DOI: 10.1107/S1600536811028649/lh5269sup1.cif

Structure factors: contains datablock(s) I. DOI: 10.1107/S1600536811028649/lh5269Isup2.hkl

Additional supplementary materials:  crystallographic information; 3D view; checkCIF report

## Figures and Tables

**Fig. 1.**
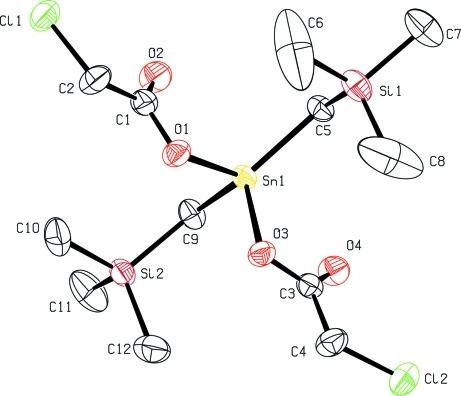
The molecular structure of title compound. Displacement ellipsoids are drawn at the 30% probability level. H atoms are not shown.

## supplementary crystallographic information

### Comment

The molecular structure of the title compound is shown in Fig. 1. The Sn^IV^ ion is coordinated in a distorted tetrahedral environment formed by two O atoms from two monodenate chloroacetato ligands and two C atoms from two trimethyl silyl ligands. There are two further weak intramolecular Sn···O contacts [2.744 (2) and 2.655 (2)Å] formed by the chloroacetato ligands. These weak contacts are also observed in a related structure (Parvez *et al.*, 1997) but are longer in the title compound.

### Experimental

A mixture of bis(trimethylsilylmethyl) diphenyltin (0.447 g, 1.0 mmol) and dichloroacetic acid (0.251 g, 2.0 mmol) were gradually heated in a oil bath to 433K the temperature was maintained 20 min. After the reaction mixture had cooled to room temperature, hexane (50 ml) was added and the mixture to dissolve the solid. Cooling the filtered solution to room temperature gave colorless crystals 0.676 g suitable for X-ray analysis, yield 96.8%.

### Refinement

All H atoms were positioned geometrically and refined using a riding model with C—H = 0.96–0.97 Å and with *U*_iso_(H) = 1.2 times *U*_eq_(C) (*U*_iso_(H) = 1.5 times *U*_eq_(C) for methyl groups). The anisotropic displacement parameters of the C atoms of the t-butyl groups are larger than normal and this might be expected. It was not considered necessary to model these as disordered atoms.

### Crystal data

**Table:**

[Sn(C_2_H_2_ClO_2_)_2_(C_4_H_11_Si)_2_]	*Z* = 2
*M**_r_* = 480.10	*F*(000) = 484
Triclinic, *P*1	*D*_x_ = 1.465 Mg m^−^^3^
Hall symbol: -P 1	Mo *K*α radiation, λ = 0.71073 Å
*a* = 10.258 (3) Å	Cell parameters from 4282 reflections
*b* = 10.767 (3) Å	θ = 2.4–26.2°
*c* = 10.808 (3) Å	µ = 1.54 mm^−^^1^
α = 71.529 (2)°	*T* = 293 K
β = 88.733 (3)°	Block, colorless
γ = 74.457 (3)°	0.28 × 0.22 × 0.17 mm
*V* = 1088.2 (5) Å^3^	

### Data collection

**Table:**

Bruker SMART CCD diffractometer	3792 independent reflections
Radiation source: fine-focus sealed tube	3311 reflections with *I* > 2σ(*I*)
graphite	*R*_int_ = 0.029
φ and ω scans	θ_max_ = 25.0°, θ_min_ = 2.1°
Absorption correction: multi-scan (*SADABS*; Bruker, 1997)	*h* = −12→12
*T*_min_ = 0.673, *T*_max_ = 0.780	*k* = −12→12
6849 measured reflections	*l* = −12→12

### Refinement

**Table:**

Refinement on *F*^2^	Primary atom site location: structure-invariant direct methods
Least-squares matrix: full	Secondary atom site location: difference Fourier map
*R*[*F*^2^ > 2σ(*F*^2^)] = 0.032	Hydrogen site location: inferred from neighbouring sites
*wR*(*F*^2^) = 0.089	H-atom parameters constrained
*S* = 1.04	*w* = 1/[σ^2^(*F*_o_^2^) + (0.0478*P*)^2^ + 0.2466*P*] where *P* = (*F*_o_^2^ + 2*F*_c_^2^)/3
3792 reflections	(Δ/σ)_max_ < 0.001
197 parameters	Δρ_max_ = 0.66 e Å^−^^3^
0 restraints	Δρ_min_ = −0.47 e Å^−^^3^

### Special details

**Table:**

Geometry. All e.s.d.'s (except the e.s.d. in the dihedral angle between two l.s. planes) are estimated using the full covariance matrix. The cell e.s.d.'s are taken into account individually in the estimation of e.s.d.'s in distances, angles and torsion angles; correlations between e.s.d.'s in cell parameters are only used when they are defined by crystal symmetry. An approximate (isotropic) treatment of cell e.s.d.'s is used for estimating e.s.d.'s involving l.s. planes.
Refinement. Refinement of *F*^2^ against ALL reflections. The weighted *R*-factor *wR* and goodness of fit *S* are based on *F*^2^, conventional *R*-factors *R* are based on *F*, with *F* set to zero for negative *F*^2^. The threshold expression of *F*^2^ > σ(*F*^2^) is used only for calculating *R*-factors(gt) *etc*. and is not relevant to the choice of reflections for refinement. *R*-factors based on *F*^2^ are statistically about twice as large as those based on *F*, and *R*- factors based on ALL data will be even larger.

### Fractional atomic coordinates and isotropic or equivalent isotropic displacement parameters (Å^2^)

**Table:**

	*x*	*y*	*z*	*U*_iso_*/*U*_eq_	
Sn1	−0.01272 (2)	0.16291 (2)	0.24712 (2)	0.04461 (12)	
Cl1	−0.12820 (13)	0.38500 (12)	−0.28104 (11)	0.0775 (3)	
Cl2	0.21959 (14)	0.16555 (14)	0.68827 (12)	0.0881 (4)	
Si1	0.30636 (12)	−0.02143 (12)	0.20680 (13)	0.0655 (3)	
Si2	−0.32332 (11)	0.38179 (11)	0.26896 (12)	0.0557 (3)	
O1	0.0062 (3)	0.3146 (3)	0.0762 (2)	0.0587 (7)	
O2	−0.1018 (3)	0.2024 (3)	−0.0036 (3)	0.0728 (8)	
O3	0.0812 (3)	0.2754 (3)	0.3262 (2)	0.0551 (6)	
O4	0.0642 (3)	0.1058 (3)	0.4965 (3)	0.0639 (7)	
C1	−0.0506 (4)	0.2954 (4)	−0.0207 (4)	0.0553 (9)	
C2	−0.0398 (5)	0.3963 (4)	−0.1488 (4)	0.0705 (12)	
H2A	0.0552	0.3833	−0.1664	0.085*	
H2B	−0.0745	0.4872	−0.1429	0.085*	
C3	0.1012 (4)	0.2083 (4)	0.4492 (4)	0.0490 (8)	
C4	0.1715 (5)	0.2691 (5)	0.5255 (4)	0.0690 (11)	
H4A	0.1115	0.3560	0.5250	0.083*	
H4B	0.2517	0.2865	0.4823	0.083*	
C5	0.1288 (4)	−0.0193 (3)	0.2482 (4)	0.0522 (9)	
H5A	0.1332	−0.0846	0.3349	0.063*	
H5B	0.0918	−0.0536	0.1881	0.063*	
C6	0.3067 (8)	0.0900 (10)	0.0410 (8)	0.273 (9)	
H6A	0.3984	0.0795	0.0158	0.409*	
H6B	0.2665	0.1829	0.0367	0.409*	
H6C	0.2555	0.0668	−0.0173	0.409*	
C7	0.4046 (5)	−0.1965 (5)	0.2236 (7)	0.1012 (18)	
H7A	0.3617	−0.2310	0.1687	0.152*	
H7B	0.4086	−0.2530	0.3130	0.152*	
H7C	0.4949	−0.1971	0.1978	0.152*	
C8	0.3879 (6)	0.0365 (10)	0.3186 (11)	0.214 (6)	
H8A	0.4825	0.0230	0.3034	0.320*	
H8B	0.3783	−0.0146	0.4073	0.320*	
H8C	0.3458	0.1316	0.3039	0.320*	
C9	−0.2193 (4)	0.2046 (4)	0.2868 (4)	0.0571 (9)	
H9A	−0.2643	0.1716	0.2309	0.069*	
H9B	−0.2242	0.1491	0.3761	0.069*	
C10	−0.3256 (6)	0.4976 (5)	0.0999 (5)	0.0943 (17)	
H10A	−0.3485	0.4573	0.0389	0.141*	
H10B	−0.2377	0.5125	0.0842	0.141*	
H10C	−0.3918	0.5831	0.0890	0.141*	
C11	−0.4989 (5)	0.3740 (6)	0.3089 (7)	0.106 (2)	
H11A	−0.5310	0.3297	0.2563	0.158*	
H11B	−0.5572	0.4647	0.2911	0.158*	
H11C	−0.4991	0.3235	0.3997	0.158*	
C12	−0.2531 (6)	0.4483 (6)	0.3835 (6)	0.0938 (17)	
H12A	−0.3184	0.5282	0.3900	0.141*	
H12B	−0.1716	0.4711	0.3516	0.141*	
H12C	−0.2329	0.3801	0.4682	0.141*	

### Atomic displacement parameters (Å^2^)

**Table:**

	*U*^11^	*U*^22^	*U*^33^	*U*^12^	*U*^13^	*U*^23^
Sn1	0.04811 (17)	0.03622 (16)	0.04990 (18)	−0.00954 (11)	0.00200 (11)	−0.01605 (11)
Cl1	0.0991 (9)	0.0731 (7)	0.0580 (6)	−0.0221 (6)	−0.0174 (6)	−0.0180 (5)
Cl2	0.1022 (9)	0.0869 (8)	0.0676 (7)	−0.0131 (7)	−0.0258 (6)	−0.0234 (6)
Si1	0.0532 (6)	0.0532 (6)	0.0860 (8)	−0.0106 (5)	0.0203 (6)	−0.0210 (6)
Si2	0.0491 (6)	0.0472 (6)	0.0674 (7)	−0.0030 (5)	−0.0021 (5)	−0.0221 (5)
O1	0.0770 (18)	0.0549 (15)	0.0437 (14)	−0.0206 (13)	0.0012 (13)	−0.0131 (12)
O2	0.099 (2)	0.0588 (18)	0.0646 (18)	−0.0337 (16)	0.0015 (16)	−0.0157 (14)
O3	0.0723 (17)	0.0488 (14)	0.0472 (15)	−0.0191 (12)	−0.0033 (12)	−0.0169 (12)
O4	0.0792 (19)	0.0548 (16)	0.0637 (17)	−0.0303 (14)	0.0067 (14)	−0.0177 (13)
C1	0.068 (2)	0.043 (2)	0.052 (2)	−0.0127 (18)	0.0034 (18)	−0.0148 (17)
C2	0.106 (3)	0.065 (3)	0.046 (2)	−0.038 (2)	−0.005 (2)	−0.0133 (19)
C3	0.049 (2)	0.0449 (19)	0.056 (2)	−0.0107 (16)	0.0041 (16)	−0.0215 (17)
C4	0.093 (3)	0.067 (3)	0.056 (2)	−0.033 (2)	−0.004 (2)	−0.022 (2)
C5	0.057 (2)	0.0384 (18)	0.059 (2)	−0.0105 (16)	0.0070 (17)	−0.0167 (16)
C6	0.148 (7)	0.238 (11)	0.212 (10)	0.066 (7)	0.128 (7)	0.121 (8)
C7	0.073 (3)	0.077 (3)	0.155 (6)	−0.008 (3)	0.024 (3)	−0.051 (4)
C8	0.067 (4)	0.272 (12)	0.420 (17)	−0.044 (5)	0.025 (7)	−0.276 (13)
C9	0.050 (2)	0.0434 (19)	0.074 (3)	−0.0112 (16)	0.0034 (18)	−0.0165 (18)
C10	0.097 (4)	0.063 (3)	0.089 (4)	0.008 (3)	−0.012 (3)	−0.004 (3)
C11	0.058 (3)	0.084 (4)	0.174 (6)	−0.010 (3)	0.024 (3)	−0.049 (4)
C12	0.095 (4)	0.094 (4)	0.106 (4)	−0.010 (3)	−0.002 (3)	−0.064 (3)

### Geometric parameters (Å, °)

**Table:**

Sn1—O1	2.088 (3)	C4—H4B	0.9700
Sn1—C5	2.102 (3)	C5—H5A	0.9700
Sn1—O3	2.108 (2)	C5—H5B	0.9700
Sn1—C9	2.108 (4)	C6—H6A	0.9600
Cl1—C2	1.762 (4)	C6—H6B	0.9600
Cl2—C4	1.755 (4)	C6—H6C	0.9600
Si1—C6	1.814 (7)	C7—H7A	0.9600
Si1—C8	1.831 (7)	C7—H7B	0.9600
Si1—C7	1.837 (5)	C7—H7C	0.9600
Si1—C5	1.862 (4)	C8—H8A	0.9600
Si2—C10	1.854 (5)	C8—H8B	0.9600
Si2—C12	1.855 (5)	C8—H8C	0.9600
Si2—C11	1.860 (5)	C9—H9A	0.9700
Si2—C9	1.871 (4)	C9—H9B	0.9700
O1—C1	1.306 (5)	C10—H10A	0.9600
O2—C1	1.214 (5)	C10—H10B	0.9600
O3—C3	1.287 (4)	C10—H10C	0.9600
O4—C3	1.217 (4)	C11—H11A	0.9600
C1—C2	1.488 (5)	C11—H11B	0.9600
C2—H2A	0.9700	C11—H11C	0.9600
C2—H2B	0.9700	C12—H12A	0.9600
C3—C4	1.500 (5)	C12—H12B	0.9600
C4—H4A	0.9700	C12—H12C	0.9600
			
O1—Sn1—C5	107.45 (13)	H5A—C5—H5B	106.9
O1—Sn1—O3	79.95 (10)	Si1—C6—H6A	109.5
C5—Sn1—O3	109.82 (13)	Si1—C6—H6B	109.5
O1—Sn1—C9	107.52 (13)	H6A—C6—H6B	109.5
C5—Sn1—C9	131.13 (15)	Si1—C6—H6C	109.5
O3—Sn1—C9	109.00 (13)	H6A—C6—H6C	109.5
C6—Si1—C8	108.8 (6)	H6B—C6—H6C	109.5
C6—Si1—C7	110.4 (4)	Si1—C7—H7A	109.5
C8—Si1—C7	107.7 (3)	Si1—C7—H7B	109.5
C6—Si1—C5	109.7 (3)	H7A—C7—H7B	109.5
C8—Si1—C5	110.8 (3)	Si1—C7—H7C	109.5
C7—Si1—C5	109.4 (2)	H7A—C7—H7C	109.5
C10—Si2—C12	108.7 (3)	H7B—C7—H7C	109.5
C10—Si2—C11	109.7 (3)	Si1—C8—H8A	109.5
C12—Si2—C11	109.7 (3)	Si1—C8—H8B	109.5
C10—Si2—C9	111.1 (2)	H8A—C8—H8B	109.5
C12—Si2—C9	110.1 (2)	Si1—C8—H8C	109.5
C11—Si2—C9	107.5 (2)	H8A—C8—H8C	109.5
C1—O1—Sn1	107.4 (2)	H8B—C8—H8C	109.5
C3—O3—Sn1	104.6 (2)	Si2—C9—Sn1	121.39 (19)
O2—C1—O1	121.8 (4)	Si2—C9—H9A	107.0
O2—C1—C2	126.1 (4)	Sn1—C9—H9A	107.0
O1—C1—C2	112.1 (3)	Si2—C9—H9B	107.0
C1—C2—Cl1	114.0 (3)	Sn1—C9—H9B	107.0
C1—C2—H2A	108.8	H9A—C9—H9B	106.7
Cl1—C2—H2A	108.8	Si2—C10—H10A	109.5
C1—C2—H2B	108.8	Si2—C10—H10B	109.5
Cl1—C2—H2B	108.8	H10A—C10—H10B	109.5
H2A—C2—H2B	107.7	Si2—C10—H10C	109.5
O4—C3—O3	121.6 (3)	H10A—C10—H10C	109.5
O4—C3—C4	124.6 (4)	H10B—C10—H10C	109.5
O3—C3—C4	113.8 (3)	Si2—C11—H11A	109.5
C3—C4—Cl2	113.7 (3)	Si2—C11—H11B	109.5
C3—C4—H4A	108.8	H11A—C11—H11B	109.5
Cl2—C4—H4A	108.8	Si2—C11—H11C	109.5
C3—C4—H4B	108.8	H11A—C11—H11C	109.5
Cl2—C4—H4B	108.8	H11B—C11—H11C	109.5
H4A—C4—H4B	107.7	Si2—C12—H12A	109.5
Si1—C5—Sn1	120.42 (18)	Si2—C12—H12B	109.5
Si1—C5—H5A	107.2	H12A—C12—H12B	109.5
Sn1—C5—H5A	107.2	Si2—C12—H12C	109.5
Si1—C5—H5B	107.2	H12A—C12—H12C	109.5
Sn1—C5—H5B	107.2	H12B—C12—H12C	109.5
			
C5—Sn1—O1—C1	75.3 (3)	O3—C3—C4—Cl2	−171.7 (3)
O3—Sn1—O1—C1	−176.9 (3)	C6—Si1—C5—Sn1	−61.1 (5)
C9—Sn1—O1—C1	−70.0 (3)	C8—Si1—C5—Sn1	59.1 (5)
O1—Sn1—O3—C3	−176.4 (2)	C7—Si1—C5—Sn1	177.7 (3)
C5—Sn1—O3—C3	−71.3 (2)	O1—Sn1—C5—Si1	45.7 (2)
C9—Sn1—O3—C3	78.4 (2)	O3—Sn1—C5—Si1	−39.7 (3)
Sn1—O1—C1—O2	−0.3 (5)	C9—Sn1—C5—Si1	179.58 (19)
Sn1—O1—C1—C2	−178.5 (3)	C10—Si2—C9—Sn1	58.6 (3)
O2—C1—C2—Cl1	7.5 (6)	C12—Si2—C9—Sn1	−62.0 (3)
O1—C1—C2—Cl1	−174.5 (3)	C11—Si2—C9—Sn1	178.6 (3)
Sn1—O3—C3—O4	−2.1 (4)	O1—Sn1—C9—Si2	−42.7 (3)
Sn1—O3—C3—C4	178.5 (3)	C5—Sn1—C9—Si2	−176.64 (19)
O4—C3—C4—Cl2	9.0 (6)	O3—Sn1—C9—Si2	42.4 (3)
